# Supplementary material for: Association between oral microbiome and five types of respiratory infections: a two-sample Mendelian randomization study in east Asian population
Source: Front Microbiol. 2024 Apr 10;15:1392473. doi: 10.3389/fmicb.2024.1392473 (PMC11039966; doi:10.3389/fmicb.2024.1392473)
Supplement: Supplementary file 1 [file Data_Sheet_1.zip › Supplementary Materials/MR plots of saliva/Chronic sinusitis/s__Veillonella_atypica_mgs_1940/leave_one_out.pdf]

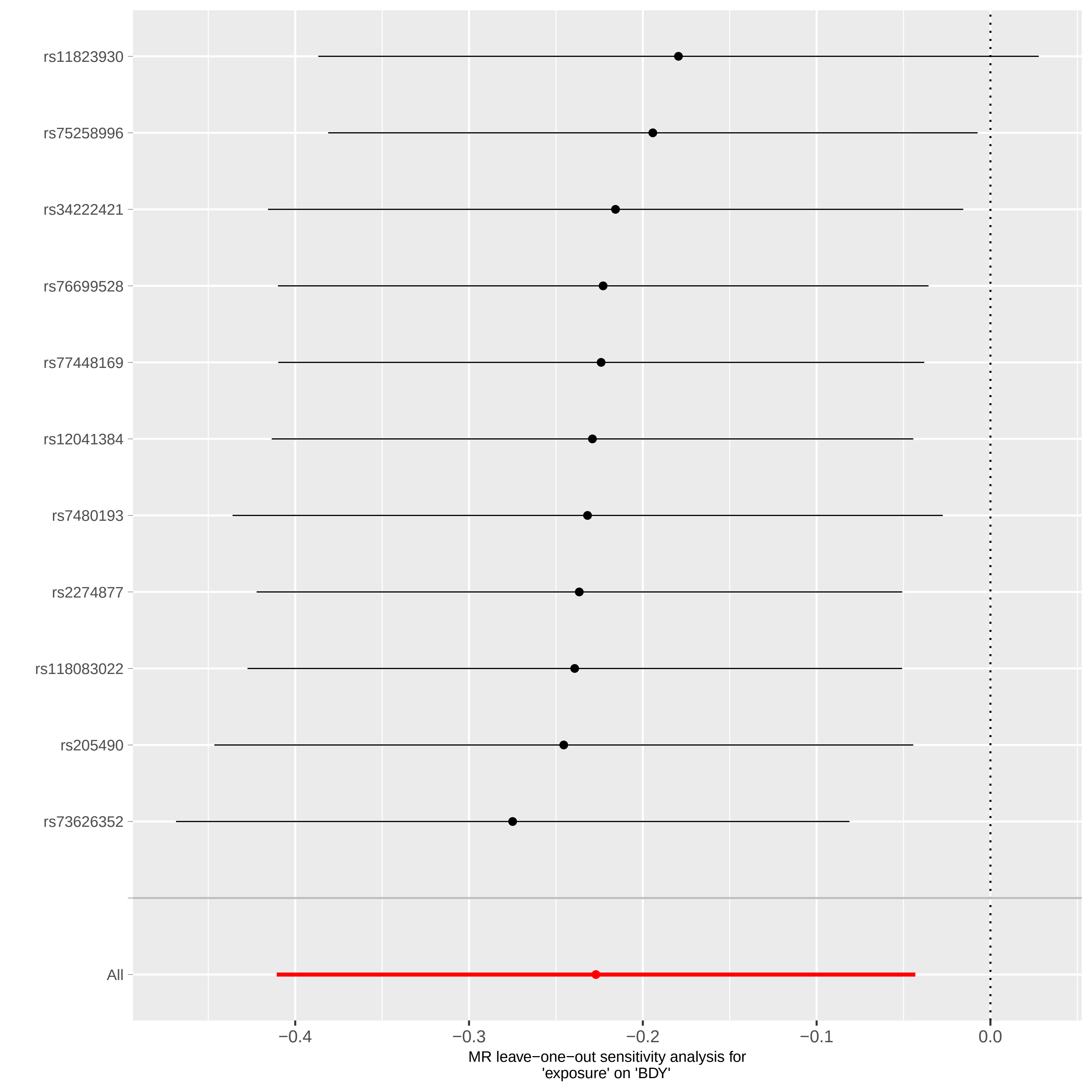
rs11823930
rs75258996
rs34222421
rs76699528
rs77448169
rs12041384
rs7480193
rs2274877
rs118083022
rs205490
rs73626352
All
−0.4
−0.3
−0.2
−0.1
0.0
MR leave−one−out sensitivity analysis for
'exposure' on 'BDY'
